# Mitochondrial Stress Signalling: HTRA2 and Parkinson's Disease

**DOI:** 10.1155/2012/607929

**Published:** 2012-05-17

**Authors:** Enrico Desideri, L. Miguel Martins

**Affiliations:** ^1^Department of Biology, University of Rome “Tor Vergata”, 00133 Rome, Italy; ^2^Cell Death Regulation Laboratory, MRC Toxicology Unit, Hodgkin Building, Lancaster Road, Leicester LE1 9HN, UK

## Abstract

Mitochondria are cellular energy generators whose activity requires a continuous supply of oxygen. Recent genetic analysis has suggested that defects in mitochondrial quality control may be key factors in the development of Parkinson's disease (PD). Mitochondria have a crucial role in supplying energy to the brain, and their deterioration can affect the function and viability of neurons, contributing to neurodegeneration. These organelles can sow the seeds of their own demise because they generate damaging oxygen-free radicals as a byproduct of their intrinsic physiological functions. Mitochondria have therefore evolved specific molecular quality control mechanisms to compensate for the action of damaging agents such as oxygen-free radicals. PTEN-induced putative kinase 1 (PINK1) and high-temperature-regulated A2 (HTRA2), a mitochondrial protease, have recently been proposed to be key modulators of mitochondrial molecular quality control. Here, we review some of the most recent advances in our understanding of mitochondria stress-control pathways, focusing on how signalling by the p38 stress kinase pathway may regulate mitochondrial stress by modulating the activity of HTRA2 via PINK1 and cyclin-dependent kinase 5 (CDK5). We also propose how defects in this pathway may contribute to PD.

## 1. Introduction

In evolutionary terms, the increase in energetic demands resulting from the evolution of small, prokaryotic organisms to larger, eukaryotic cells was achieved through the internalisation of energy-generating factories that ultimately led to the emergence of modern mitochondria [1]. Such an increase in energy output also resulted in detrimental consequences because these organelles are the main intracellular sources of damaging oxygen-free radicals. These reactive oxygen species (ROS) can be destructive, attacking various cellular components, including DNA, proteins, lipids, and carbohydrates, but can also act as regulators of intracellular signalling pathways [2]. Eukaryotic cells have evolved strategies to cope with the damage caused by excess levels of damaging agents such as ROS. Recent findings from genetic studies suggest that the defective sensing of mitochondrial damage may play an important role in the development of neurodegenerative diseases such as Parkinson's disease (PD) (reviewed in [3]). An effective response to such damage can be described in three different steps: (1) damaged components need to be recognised by sensing mechanisms, (2) the sensing mechanisms must convey a signal to damage suppressors, and (3) the activity of damage suppressors must be increased to promote the disposal of damaged cellular components. 

In this paper, we provide an overview of the research that focuses on how pathways affecting mitochondrial quality control may play a role in the aetiology of PD. We place particular emphasis on a recently identified mitochondrial damage response pathway regulated by p38 kinase and summarise some of the recent molecular determinants of mitochondrial quality control regulated by this kinase.

## 2. Parkinson's Disease and Mitochondrial Dysfunction

PD is a common neurodegenerative disease characterised by the progressive loss of dopaminergic neurons in the nigrostriatal region of the brain. Most PD cases occur sporadically (i.e., they are of unknown cause). However, 10–15% of PD patients have a family history of the disease, indicating that there is a strong genetic basis for this disease in this subgroup. The molecular pathogenesis of sporadic PD and the basis for selective dopaminergic neuron loss remain unknown, and it is unclear whether gene mutations are involved in the development of this disease in sporadic PD patients. Epidemiological studies consistently link exposure to pesticides to a higher incidence of PD. In particular, pesticides that cause an increase in ROS, such as rotenone and paraquat, have been shown to cause PD-like conditions in rodent models [4]. In addition, the mitochondrial toxin 1-methyl-4-phenyl-1,2,3,6-tetrahydropyridine (MPTP) was shown to be responsible for the onset of severe PD-like symptoms in a group of young drug users in the 1980s. Drugs such as rotenone, paraquat, and MPTP disrupt normal electron transport chain (ETC) in the mitochondria, leading to an increase in free radical generation. Through the generation of ROS, exposure to mitochondria-damaging agents may be important in the aetiology of PD in sporadic patients. Although mitochondrial dysfunction has been inconclusively linked to PD in the past few decades, genetic evidence indicating mitochondrial involvement in this disease was recently obtained. A major advance occurred in a recent study in which researchers identified disease-causing *PINK1* mutations in familial PD [5]. Mutations in the mitochondrial serine protease *HTRA2* were also reported to be associated with PD in sporadic patients [6]; however, the role of HTRA2 in PD remains controversial [7].

## 3. Mitochondria as the Major Source of Intracellular ROS

Mitochondria are the powerhouses of eukaryotic cells and are responsible for most of the ATP synthesis via oxidative phosphorylation (OXPHOS). During OXPHOS, NADH, and FADH_2_ produced by glycolysis and the tricarboxylic acid (TCA) cycle are used as electron donors and transported through the ETC via a series of redox reactions that involve four molecular complexes (Complexes I, II, III, and IV). Oxygen is used as the final electron acceptor and is reduced to water through the acquisition of four electrons. The transport of electrons through the ETC is coupled to the discharge of protons from the mitochondrial matrix to the intermembrane space. The discharge of protons leads to the generation of a proton gradient that is necessary for the synthesis of ATP from ADP by ATP synthase. During transport through the ETC, a small portion of electrons (1–3%) escape prematurely, mainly from Complexes I and III, and directly reduce oxygen, generating the superoxide anion (O_2_^•−^). These ROS can interact with other molecules to form other types of ROS, such as hydrogen peroxide (H_2_O_2_) and the hydroxyl radical (OH^•^). In addition to the ETC, other mitochondrial components are known to generate ROS, such as *α*-ketoglutarate dehydrogenase (*α*-KGDH) and pyruvate dehydrogenase, which generate both the superoxide anion (O_2_^•−^) and hydrogen peroxide (H_2_O_2_) [8, 9]. In addition to the physiological ROS produced by normal mitochondrial activity, nonphysiological increases in ROS levels can occur in conditions of stress, such as nutrient deprivation or hypoxia, or as a consequence of the deterioration of mitochondrial enzymes. The increase in ROS concentration, a condition known as oxidative stress, can be detrimental for the mitochondria and the entire cell because of the capacity of ROS to damage several cellular components, including proteins, lipids, and nucleic acids. To cope with the detrimental effects of ROS, mitochondria are equipped with several antioxidant systems that comprise a first line of defence against these toxic agents. Of these systems, the most important is manganese SOD (Mn-SOD). Mn-SOD is a highly efficient enzyme (*K*_cat_ ~ 10^9^ M^−1^ sec^−1^) localised to the mitochondrial matrix that can quickly dismutate superoxide anions (O_2_^•−^) to hydrogen peroxide (H_2_O_2_) and molecular oxygen (O_2_). In addition to Mn-SOD, mitochondrial antioxidant defences include the thioredoxin-2 (Trx2) system, formed by Trx2 and thioredoxin reductase 2 (TrxR2), and peroxiredoxin-3 (Prx3); both are involved in the scavenging of hydrogen peroxide (reviewed in [10]).

## 4. Mitogen-Activated Protein Kinases Are Mediators of ROS-Linked Signal Transduction

Paradoxically, even though mitochondria-generated ROS are clearly viewed as damaging agents, they can also play an active role in intracellular signalling. Hydrogen peroxide, but not the superoxide anion, can easily cross the mitochondrial membranes and diffuse into the cytosol, where it can negatively affect the structure and function of proteins by the specific oxidation of reactive cysteine residues to sulphenic acid (–SOH) (reviewed in [11]). Sulphenic acid is a very reactive and unstable chemical and, together with ROS, can further react with a second molecule of H_2_O_2_ to form a sulfinic acid derivative (–SO_2_H). Oxidation to sulfinic acid is, with some exceptions, an irreversible modification that can permanently alter the structure and function of a protein and can ultimately result in cellular damage and death. Under physiological conditions, sulphenic acid derivatives can be reduced back to the thiolate form or transformed into a number of thiol adducts by reactions such as *S*-glutathionylation to form protein-GSH mixed disulphide, a stable and reversible oxidation state that can be efficiently reduced back by reactions catalysed by Trx and peroxiredoxins. The reversible oxidation of cysteines by ROS, in particular H_2_O_2_, allows these molecules to be classified as second messengers, which lead to the activation of signalling pathways. Of these, pathways mediated by mitogen-activated protein kinases (MAPKs) are some of the best studied and characterised.

MAPKs are a family of evolutionarily conserved serine/threonine kinases involved in the regulation of several cellular processes, such as growth, differentiation, and apoptosis. A typical MAPK cascade includes a MAPK kinase kinase (MAP3K) that phosphorylates a MAPK kinase (MAP2K), which in turn phosphorylates a MAPK. Active MAPKs, through direct phosphorylation, regulate the activity of many cytoplasmic and nuclear targets. The switch from a redox signal to a phosphorylation cascade can occur at different levels of the MAPK pathway, such as at the level of the MAP3Ks, some of which (e.g., ASK1 and MEKK1) are redox-sensitive proteins. ASK1, in particular, plays a key role in the cellular response to oxidative stress because it can activate both JNK and p38 pathways through the phosphorylation of their upstream kinases MKK4/7 and MKK3/6, respectively [12]. The activation of ASK1 is regulated by its redox-sensitive binding with thioredoxin-1 (Trx1), an antioxidant protein involved in the reduction of disulphide bonds. Under physiological conditions, one of the two cysteines in the active site of Trx1 binds and inactivates ASK1; when oxidative stress occurs, the Trx1 cysteines form an intramolecular disulphide bond, and the protein loses its interaction with ASK1, which becomes active and can activate downstream factors [13, 14]. The ultimate effect of MAPK activation can range from cell proliferation to cell death and is strongly influenced by the duration and magnitude of their direct phosphorylation. MAPK phosphorylation depends on an equilibrium state between the activity of upstream kinases and specific MAPK phosphatases (MKPs). MKPs are a group of protein phosphatases, including tyrosine, serine/threonine, and dual-specificity MAPK phosphatases, that dephosphorylate MAPKs and block the signalling pathways on which they depend. ROS, in addition to being able to promote MAPK phosphorylation, are known to inhibit the activity of MKPs through the reversible oxidation of their reactive cysteine residues, thus contributing to a prolonged activation of MAPKs [15]. There are three main MAPK cascades in mammals: those mediated by the extracellular signal-regulated kinases (ERK1/2), the c-Jun NH_2_-terminal kinases or stress-activated kinases (JNK/SAPK) and p38. The ERK cascade is mainly involved in the control of cell proliferation and differentiation, whereas the p38 and JNK pathways are implicated in the control of cell survival and cell death because they are activated by environmental stresses, which are often associated with the generation of ROS [16, 17].

## 5. Modulation of Quality Control in Mitochondria

Numerous findings have suggested that disruptions in mitochondrial function and dynamics contribute to ageing and neurodegenerative diseases (reviewed in [22]). Cells have therefore developed molecular mechanisms to cope with the diverse challenges imposed on mitochondrial integrity. Mitochondria are thought to have at least two levels of defence mechanisms that ensure their integrity and viability in individual cells (reviewed in [23]). The first line of defence comprises highly specific molecular quality control machinery, including molecular chaperones and proteases that monitor the folding and assembly of mitochondrial proteins. Interestingly, both PINK1 and HTRA2 seem to be important modulators of molecular quality control in mitochondria. The deletion of *HTRA2* from mice results in an increase in ROS levels and an accumulation of misfolded proteins in brain mitochondria [24], and an analysis of postmortem brain tissue obtained from PD patients with mutations in *PINK1* revealed an increase in the levels of misfolded mitochondrial respiratory complexes in the brain [25].

Once mitochondrial molecular quality control is overwhelmed, a second line of defence, termed organellar quality control, is thought to take over. Organellar quality control relies on the dynamic nature of mitochondrial populations to ensure the disposal of defective mitochondrial components via mitochondrial fission and autophagy (reviewed in [3]). Mitochondrial dynamics are thought to be important for the control of mitochondrial turnover and bioenergetic efficiency. The combined functions of fusion, fission, and autophagy are now emerging as essential organellar quality control mechanisms that promote the sequestration, sorting, and elimination of functionally impaired mitochondria [26]. PINK1 seems to play a key role in organellar quality control. PINK1 is capable of recruiting parkin, a cytosolic ubiquitin ligase, to damaged mitochondria and targeting these organelles for autophagic clearance [27].

If both molecular and organellar quality control mechanisms fail, severe mitochondrial damage can lead to the uncontrolled release of mitochondrial proteins including cytochrome c. Once it reaches the cytosol, cytochrome c unleashes the apoptosis pathway of cell death (reviewed in [28]).

It is conceivable that a failure of either one of these quality control mechanisms in mitochondria ultimately results in the demise of dopaminergic neurons observed in PD and therefore plays a causative role in this disease.

## 6. Activation of Mitochondrial Damage Responses by the p38 Stress Kinase

As stated above, it is conceivable that upon mitochondrial damage, intracellular mechanisms act to convey a signal to damage suppressors to attempt to counteract such damage. In this context, recent evidence suggests that the p38 stress kinase may fit the role of such an intracellular sensor mechanism by conveying signals to mitochondrial proteins, such as the putative kinase PINK1 and the protease HTRA2, to activate mitochondrial quality control defence mechanisms. Disease-causing mutations in *PINK1* are linked to familial PD [5], whereas *HTRA2* mutations were reported to be present in sporadic PD patients [6, 19].

A role for HTRA2 as a proapoptotic factor was initially described by several groups [29–32]. However, more recently, *in vivo* studies in mice with a loss-of-function mutation in the *HTRA2* gene (S276C) and in *HTRA2* knockout mice showed that these animals are characterised by a lethal neurodegenerative disorder [33, 34] with an accumulation of unfolded proteins in the mitochondria [24]. This indicates that the activity of mitochondrial HTRA2 protease may be important for controlling the levels of misfolded proteins in mitochondria in a manner similar to its bacterial homologues DegP and DegS [35, 36]. As with many other proteases, the proteolytic activity of HTRA2 is tightly regulated to prevent unwanted proteolysis. Structural studies have shown that interactions between its protease domain and its regulatory PDZ domain keep the proteolytic activity of HTRA2 in check until the PDZ domain is engaged by binding to C-terminal PDZ-binding peptides or internal hydrophobic stretches in misfolded proteins [35, 36]. The activation of HTRA2 has also been shown to occur via the direct phosphorylation of S142 through an interaction with PINK1 [20]. This PINK1-mediated phosphorylation promoted by p38 results in an increase in HTRA2 proteolytic activity, which, in turn, increases its protective effects in mitochondria.

More recently, a novel phosphorylation site (S400) has been identified in HTRA2 [21]. This site lies in the PDZ domain of the protease, a region known to modulate its protease function [37], which may also be involved in neuroprotection [20]. Phosphorylation of HTRA2 at S400 is promoted by CDK5, a serine/threonine kinase that is a member of the highly conserved family of cyclin-dependent kinases. This kinase is unique among its family members because it is neither activated by cyclins nor regulates the cell cycle (reviewed in Dhavan and Tsai [38]). Mice lacking *CDK*5 die prematurely and demonstrate a disruption in neuronal layering [39]. Abnormal CDK5 activity is associated with several neurodegenerative diseases. In particular, CDK5 accumulates in neurons in Lewy bodies, the principal hallmark of PD. CDK5-dependent HTRA2 phosphorylation via the p38 pathway is involved in maintaining the mitochondrial membrane potential under stressful conditions and results in protection against cellular stress [21].

**Figure 1:**
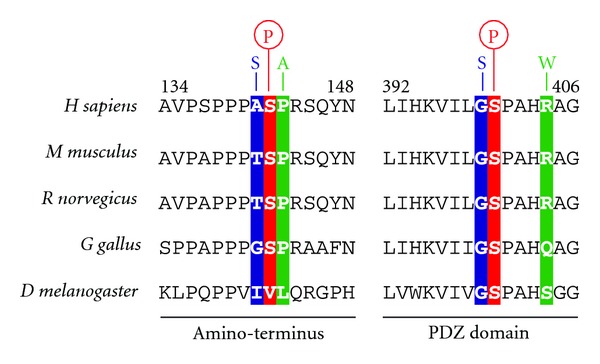
Mutations in *HTRA2* associated with Parkinson's disease lie in close proximity to p38 and CDK5 phosphorylation sites. Mutations in the amino-terminus (A141S [6] and P143A [18]) and the PDZ domain (G399S [6] and R404W [19]) are indicated. The relative positions of the phosphorylation sites in human HTRA2 (see [20, 21]) are indicated by the circles above the serine residues. A sequence alignment with *HTRA2* orthologues from several species is shown.

Curiously, the mutations in the *HTRA2* gene found in PD patients seem to lead to amino acid changes in residues that lie in close proximity to the identified phosphorylation sites in this protease. Both the A141S [6] and the P143A [18] mutations found in PD patients are near S142, whereas G399S [6] and R404W [19] are near the S400 phosphorylation site (Figure 1). These findings suggest that such mutations may affect the phosphorylation status of HTRA2 and therefore have a detrimental role in the activation of this enzyme downstream of p38 signalling.

## 7. Concluding Remarks

**Figure 2:**
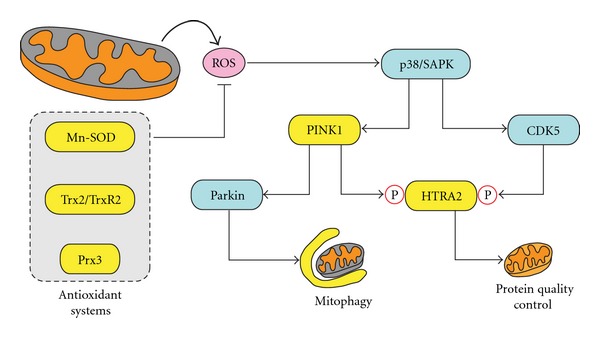
Modulation of mitochondrial quality control by the p38 stress kinase. When mitochondrial stress occurs, ROS production results in the activation of p38. Through its downstream effectors, PINK1 and CDK5, this pathway results in an increase in the proteolytic activity of mitochondrial HTRA2, which in turn contributes to the suppression of mitochondrial damage by enhancing protein quality control. By promoting the recruitment of cytosolic parkin to damaged mitochondria, PINK1 can also contribute to the clearance of these organelles through mitophagy. Antioxidant systems modulate the levels of ROS and can therefore affect signalling from p38 to either PINK1 or CDK5. Yellow: mitochondrial proteins; cyan: cytosolic proteins.

Taken together, the findings outlined in this paper are suggestive of a role for the p38 pathway in modulating molecular quality control in mitochondria. It is conceivable that following mitochondrial stress, mild ROS production results in the activation of a mitochondrial damage-sensing mechanism involving p38. This activation conveys a signal to downstream damage suppressors via PINK1 and CDK5, resulting in an increase in the proteolytic activity of mitochondrial HTRA2, which then contributes to the suppression of mitochondrial damage by increasing the disposal of damaged mitochondrial components, such as misfolded proteins (Figure 2). Failure to activate such a pathway would result in an accumulation of mitochondrial damage and perhaps result in overwhelming levels of ROS. Such high levels of ROS would ultimately lead to the loss of integrity of the mitochondrial membranes, causing the release of proapoptotic proteins and eventual cell death.

Clearly, protein misfolding plays an important role in the development of PD. Although clear mechanisms for such protein misfolding pathologies are well established when accumulation occurs in the cytosol, cell nucleus, endoplasmic reticulum, and extracellular space, little is known about any causative role of protein aggregation in the mitochondria in PD.

Neurodegenerative diseases are a group of adult-onset pathologies of increasing clinical interest, considering the growing number of new cases each year and the increasing human lifespan, as age is one of the main risk factors. After many years of research, the crucial role of mitochondria in neurodegeneration has been established and is now widely accepted. Mutations of mitochondrial proteins or proteins involved in mitochondrial function have been found in familiar cases of such diseases, but the role of many of those proteins is still unclear. For example, HTRA2 is strongly implicated in neurodegeneration, in particular PD, but this has not been accompanied by a complete characterisation of its activity. A deeper comprehension of the role of the proteins involved in the control of mitochondrial homeostasis could provide better knowledge of the molecular mechanisms underlying the development of neurodegenerative disorders associated with mitochondrial dysfunction and could help the development of novel strategies able to effectively block the course of the disease.

## Abbreviations

ROS: Reactive oxygen species
OXPHOS: Oxidative phosphorylation
ETC: Electron transport chain
ATP: Adenosine triphosphate
NAD: Nicotinamide adenine dinucleotide
FAD: Flavin adenine dinucleotide
TCA: Tricarboxylic acid
SOD: Superoxide dismutase
MPTP: 1-Methyl-4-phenyl-1,2,3,6 tetrahydropyridine.
